# Microfluidic Passive Valve with Ultra-Low Threshold Pressure for High-Throughput Liquid Delivery

**DOI:** 10.3390/mi10120798

**Published:** 2019-11-21

**Authors:** Xinjie Zhang, Ayobami Elisha Oseyemi

**Affiliations:** 1 College of Mechanical and Electrical Engineering, Hohai University, Changzhou 213022, China; ae.oseyemi@hhu.edu.cn; 2 School of Mechanical Engineering, and Jiangsu Key Laboratory for Design and Manufacture of Micro-Nano Biomedical Instruments, Southeast University, Nanjing 211189, China

**Keywords:** microfluidic, passive valve, ultra-low pressure, flow control, high flow rate

## Abstract

The microvalve for accurate flow control under low fluidic pressure is vital in cost-effective and miniaturized microfluidic devices. This paper proposes a novel microfluidic passive valve comprising of a liquid chamber, an elastic membrane, and an ellipsoidal control chamber, which actualizes a high flow rate control under an ultra-low threshold pressure. A prototype of the microvalve was fabricated by 3D printing and UV laser-cutting technologies and was tested under static and time-dependent pressure conditions. The prototype microvalve showed a nearly constant flow rate of 4.03 mL/min, with a variation of ~4.22% under the inlet liquid pressures varied from 6 kPa to 12 kPa. In addition, the microvalve could stabilize the flow rate of liquid under the time-varying sinusoidal pressures or the square wave pressures. To validate the functionality of the microvalve, the prototype microvalve was applied in a gas-driven flow system which employed an air blower or human mouth blowing as the low-cost gas source. The microvalve was demonstrated to successfully regulate the steady flow delivery in the system under the low driving pressures produced by the above gas sources. We believe that this new microfluidic passive valve will be suitable for controlling fluid flow in portable microfluidic devices or systems of wider applications.

## 1. Introduction

The conceptual design of microfluidics or lab-on-a-chip associated with point-of-care test (POCT) applications, such as biological cell separation [1,2], nucleic acid diagnostic [3,4], bacteria detection, etc. [5,6] usually require high-throughput processing, autonomous actuation, and portability. As a key component of microfluidic POCT systems, a micro-scaled pumping unit for driving liquid should meet the technical functionality of the system. Nowadays, microvalves are being employed widely in pumping units for sophisticated flow control, and valve structure and actuation mechanism are key factors influencing their utility in limited resource applications. Generally, the geometry of the microvalve should be designed for a potential large-scale integration. The overall force for controlling liquid in the microvalve should be minimized for convenient actuation. In addition, for the sake of cost-effectiveness and portability, the microvalve should be wholly actuated on-chip with energy efficiency.

Many microvalves with the functionality of on-chip actuation have been reported in previous literatures, especially the elastomeric membrane types which have been recognized as a cornerstone technology for enabling various applications in microfluidic systems [7,8]. These membrane valves are flexible in actuation, easy to manufacture by standard fabrication processes, and feasible for miniaturized integration with microfluidic devices for biological and biochemical applications. A typical membrane valve in structure would have three layers, including a control channel, a fluidic channel, and a thin elastic membrane, which can reversibly deflect to open or close the fluidic channel for flow control [9]. Generally, poly(dimethylsiloxane) (PDMS) is the most commonly used membrane material due to its good optical transparency and high elasticity for large deformations [10]. Other materials, such as thermal plastic polymer [11,12], shape memory alloy [13,14], glass [15], and more are also used in certain situations. As for the deflection of the membrane, various mechanical [16,17], electrostatic [18,19], pneumatic [20,21], magnetic [22,23], piezoelectric [24], or thermal [25] mechanisms have been proposed. Among the actuation mechanisms, pneumatic actuation is apparently the most commonly used technology. An active valve with pneumatic actuation usually employs off-chip apparatus such as an air-compressor, a pressure regulator, etc., and does the required flow regulation by the regulation of the air pressure. The valve can be combined to form complex microfluidic devices, such as peristaltic pumps [11,19] and mixers [26]. However, when a low-cost and portable microfluidic system such as in POCT applications requires totally on-chip activation, the active technology may be considered excessive. An alternative technology for liquid control is the passive actuation. In comparison to the active valve, a passive valve doesn’t require any external power, and it regulates the flow rate of liquid through autonomous adjustment of flow resistance. In addition, as it is capable of realizing self-adaptive resistance variations which completely compensate the fluidic pressure variations, a constant flow rate can, as a result, be achieved by the valve with a pre-determined threshold pressure [27,28,29]. Cousseau et al. developed a silicon membrane valve for drug delivery [30]. The valve comprised of a glass cover, a silicon membrane, and a bottom layer with a spiral channel. The valve was able to provide a constant liquid flow rate of 0.022 mL/min within an operating pressure range of 20 kPa to 50 kPa. Kartalov et al. proposed a PDMS push-up valve which was composed of a detour control channel, a membrane, and a fluidic channel [31]. The constant flow rate maintained by the valve was 0.033 mL/min, and the threshold pressure to achieve the flow rate was 103 kPa. Yang et al. designed a compliant flap and a rigid stopper which formed a restricted fluid path in a planar check valve, and the valve output a high flow rate of 1.2 mL/min with a threshold pressure of 100 kPa [32]. For fabricating passive valve with low threshold pressure, Doh et al. presented a parallel membrane valve which included two control channels, two vertical membranes, and a fluidic channel [33]. As the two membranes could restrict liquid in the fluidic channel with the autonomous deflection, the valve was capable of achieving flow regulation at a minimum pressure of 15 kPa. Our team had previously reported a parallel membrane valve with two horizontal membranes sandwiching a fluidic channel between them, and then all sandwiched between two control channels [34,35]. Due to the five-layer stacked architecture, the valve achieved a high flow rate of 2.79 mL/min with a low threshold pressure of 10 kPa. Thus, the valve could be used for high throughput sample processing in biological cell separation [36,37]. However, these existing passive valves can be difficult to integrate into useful microfluidic due to their high threshold pressures for flow regulation. For example, most mechanical and non-mechanical micropumps generate fluidic pressures lower than 10 kPa [38,39], which leaves the current valves with a major challenge when it comes to integrating them for such a low-pressure flow regulation.

In this work, we developed a new microfluidic passive valve which can produce a constant flow rate of liquid under an ultra-low threshold pressure. To investigate the flow characteristics of the valve, we measured the flow rates of the valve under static and the time-dependent conditions of a varied set of inlet pressures. We also demonstrated some smart function abilities of the valve in combination with a gas-driven flow system for obtaining high throughput and stable flow delivery under low driving pressures.

## 2. Materials and Methods 

### 2.1. Working Principle

**Figure 1:**
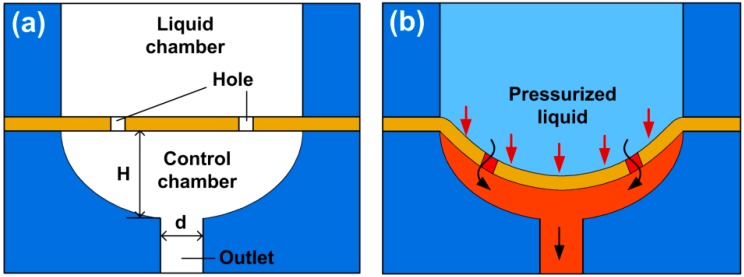
Working principle of the microfluidic passive valve. (**a**) Concept structure of the valve. (**b**) Schematic valve actuation under pressurized liquid.

The structure schematic of the proposed passive valve concept is shown in Figure 1a. The valve comprises of a liquid chamber, an elastic membrane, and a control chamber. The liquid chamber connects to the valve inlet for liquid input. The control chamber has an ellipsoid surface, which connects to the valve outlet for liquid output. Two micro-sized holes were designed in the membrane for liquid to flow through, while the membrane would deform towards the control chamber under liquid pressure to regulate the outlet flow rate. As described in Figure 1b, when the inlet liquid pressure is increased to deflect the membrane, the volume of the control chamber decreases due to the membrane deformation, hence the flow resistance of the valve increases. The flow rate of the valve can be estimated as:

(1) $$Q=\frac{P}{R}=\frac{P+\Delta P}{R+\Delta R}$$

where *P* is the initial inlet pressure, Δ*P* is the pressure gradient, *R* is the initial flow resistance of the valve, and Δ*R* is the resistance increment. From Equation (1), when the inlet pressure is increased beyond a threshold value, the resistance increment Δ*R* automatically compensates for the pressure change Δ*P*, thus a consistent flow rate *Q* is obtained over a range of increased pressures. 

**Figure 2:**
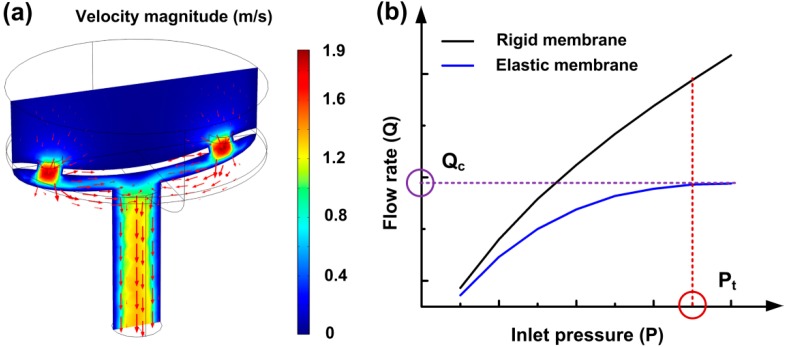
Fluid-Structure Interaction simulation of valve actuation. (**a**) Simulated velocity magnitude in the valve. (**b**) Simulated flow rate versus the inlet pressure in the valve.

Based on the aforementioned concept, we conducted a numerical study on the valve using Fluid-Structure Interaction (FSI) module in COMSOL Multiphysics^®^ (Version 4.3b, COMSOL Inc., Stockholm, Sweden), which allowed us to investigate the two-way interaction between the elastic membrane and the liquid (Figure 2a). In the FSI model, the liquid domain was built on the incompressible Navier-Stokes model, taking the dynamic viscosity of water as 0.001 Pa∙s, while the solid domain was built using a solid stress-strain model for PDMS membrane with a Young’s modulus of 1.7 MPa and a Poisson’s ratio of 0.49 [33]. The simulation was performed across a range of increased inlet pressure, up to the point where the membrane made contact with the wall of the control chamber. In the simulation, a significant nonlinear relationship was observed between the flow rate and the inlet pressure, which is attributable to the self-adaptive resistance variation characteristic of the valve as illustrated in Figure 2b. At first, the membrane slightly deformed with the increase of the inlet pressure, which led to a high slope of the Q(P) curve. When the inlet pressure was increased to a certain threshold value of P_t_, the membrane deformed dramatically to approach the wall of the control chamber, inducing a highly increased flow resistance in the control chamber. At this moment, the volume of the control chamber was saturated at a certain non-zero value, thereby generating a corresponding resistance gradient to compensate the increased pressure. After that, the slope of the Q(P) curve became very small and went down to zero gradually. Finally, the flow rate across the valve was regulated to near-constancy. Also, we solved the model using a rigid membrane (rather than a flexible as in the original case), and the outcome was a continuously increasing flow rate proportionate to the inlet pressure increase. Comparing the simulation results of the two models, we could estimate the feasibility of the flow autoregulation by the proposed valve from theoretical insight.

### 2.2. Valve Design

**Figure 3:**
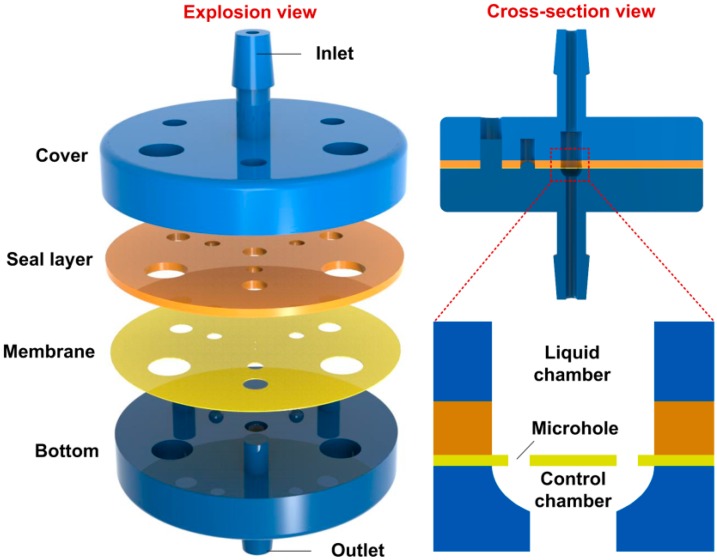
Schematic 3D architecture of the microfluidic passive valve. The explosion view shows the four parts of the valve, and the cross-section view shows the inner structure of the valve.

The 3D architecture of the valve is shown in Figure 3. The valve is composed of four functional parts: the cover, the seal layer, the membrane, and the bottom (see the explosion view of the valve). The cover has the inlet while the bottom has the outlet, with diameters 800 µm and 600 µm, respectively. Two through holes with a diameter of 1500 μm were designed in the cover and the seal layer to form the liquid chamber (see the cross-sectional view of the valve). Two micro-holes, each with a diameter of 200 µm were designed in the membrane with a Centre Distance of 1000 µm between them. The seal layer and the membrane are of 500 µm and 50 µm thickness, respectively. The bottom was designed with a control chamber with an ellipsoid surface, 1500 µm in diameter, and 150 µm in depth. We also designed some orientation pillars and holes in each part for ease and accuracy of assembly.

### 2.3. Device Fabrication

**Figure 4:**
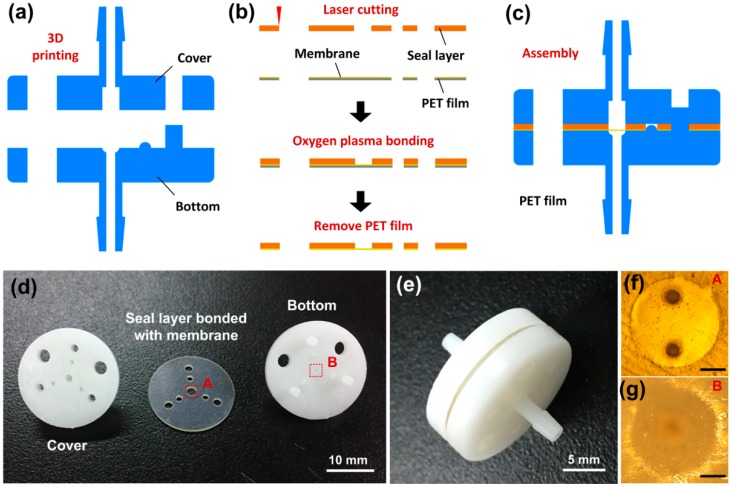
Fabrication of the microfluidic passive valve. Schematics of the valve fabrication, (**a**) 3D printing of the cover and the bottom, (**b**) UV laser micromachining of the seal layer and the membrane, and (**c**) assembly of the valve. (**d**) Photograph showing the prototype functional parts of the valve. (**e**) Photograph showing a prototype of the assembled valve. (**f**) Image showing the micro-holes in the membrane at position A in Figure 4d. (**g**) Image showing the ellipsoid surface of the bottom at position B in Figure 4d. Scale bar in Figure 4f,g was 500 μm.

A multi-layer fabrication method was developed to build the functional parts of the valve. The cover and the bottom (SOMOS Imagine 8000, DSM, Shanghai, China) were designed in the commercial CAD software SolidWorks (SolidWorks 2016, Dassault Systems SolidWorks Corporation, Waltham, MA, USA) and then produced on a 3D printer (Figure 4a). The seal layer and the membrane were fabricated using silicon film and PDMS film, respectively. The seal layer and the membrane were designed in AutoCAD software (AutoCAD 2013, Autodesk Inc., San Rafael, CA, USA) and transferred to a UV laser machine (AWAVE 355-10W-30K, Advanced Optowave Corporation, Suffolk, NY, USA) for cutting and engraving. As the PDMS film was adhered to a layer of PET (Polyethylene terephthalate) film, the silicon film was at first bonded with the PDMS film using oxygen plasma treatment, after which the PET film was gently removed from the surface of the two bonded films (Figure 4b). The 3D printed-parts and laser-cut films were washed using pure ethanol, followed by rinsing using deionized water and air-dry. Finally, the cover, the bonded seal layer, and the membrane, and the bottom were assembled together layer by layer through the orientation pillars and holes (Figure 4c). The fabricated prototypes of the functional parts and the valve are shown in Figure 4d,e. The UV laser technology used to cut out the micro-holes in the membrane gave them smooth edges with precision of size (Figure 4f). The ellipsoid surface was factored into the bottom cover design, and so it was generated by 3D printing (Figure 4g).

### 2.4. Experimental Setup

**Figure 5:**
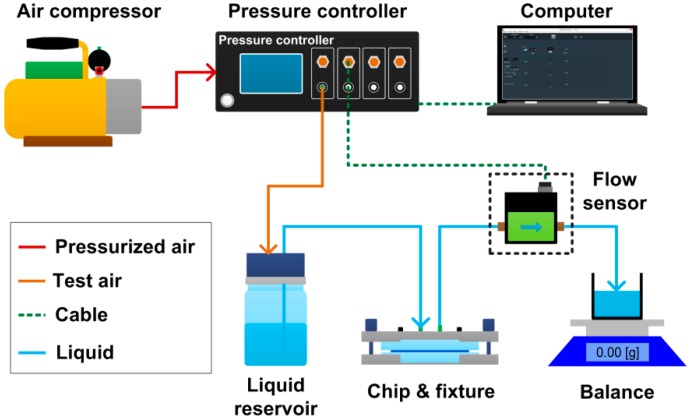
Schematic diagram of the experimental setup for flow rate measurement in the microfluidic passive valve.

A set of flow measurement setup was used to measure the flow rate of the microfluidic passive valve under a set of target test pressures. As shown in Figure 5, an air compressor was used to supply compressed air (CDA) to a pressure controller (OB1 Base MkIII, Elveflow, Paris, France). The pressure controller regulated the CDA to a target pressure and output the pressurized air into a sealed reservoir which was filled with deionized water. The deionized water was then pushed out to flow through the valve which was held by a fixture, and flow rate across the valve was monitored with a flow sensor (MFS 5, Elveflow, Paris, France). As the pressure controller was connected to the flow sensor and a computer for data interaction, the flow rates under varied test pressures could be recorded by the computer. For the measurement of flow rate higher than the upper limit of the flow sensor, an electronic balance (AX523ZH, OHAUS, Parsippany, NJ, USA) was used to replace the flow sensor, which measured the accumulated mass change of the liquid during one minute. 

## 3. Results and Discussion

### 3.1. Flow Characterization Under Static Pressure

**Figure 6:**
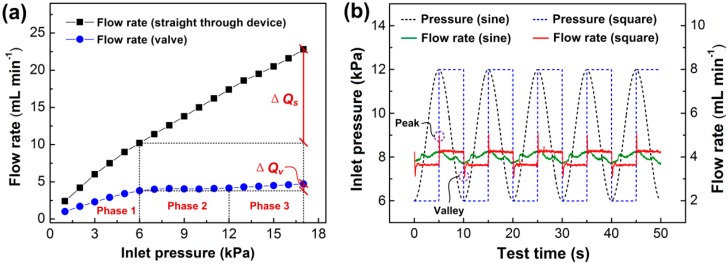
Flow rates of the microfluidic passive valve under varied inlet pressures. (**a**) Valve flow rate under static pressures. Δ*Qs* and Δ*Qv* represent the increments of flow rates under inlet pressures from 6 kPa to 17 kPa, respectively. (**b**) Valve flow rate under dynamic inlet pressures. The black and blue dash lines represent the sinusoidal pressure and the square pressure, while the green and red solid lines represent the flow rates of the valve under the above two pressures, respectively.

As previously mentioned, the working principle of the valve is such that flow rate across the valve is auto-regulated by the deformations of the membrane. To investigate the flow characteristics of the valve, we measured the flow rates of the prototype valve at different inlet liquid pressures, and the corresponding effects of varied pressures on the flow rates were analyzed accordingly. Flow tests were at first performed under statically varied pressures as shown in Figure 6a. In the experiment, the inlet liquid pressure of the valve was increased sequentially from 1 kPa to 17 kPa by 1 kPa step. The flow rate at each test pressure was measured and recorded in a minute. To quantitatively study the effect of the inlet pressure on the flow rate, we divided the flow rate curve into three phases based on the flow performances induced by the inlet pressures. In the first phase, the flow rate was in direct proportionality to the inlet pressure, and it increased steadily as the pressure increased from 1 kPa to 6 kPa. When the pressure was higher than 6 kPa, the flow rate began to show a significant nonlinear relationship with the inlet pressure. In the 6 kPa to 12 kPa pressure phase, we found that the flow rate was getting regulated, as it maintained a nearly constant value regardless of pressure change. To analyze the functionality of the valve, we calculated the mean flow rate and the flow variation across the above pressure ranges. The flow variation defined as the relative pulsation to the mean flow rate was calculated as a ratio of the bilateral tolerance of minimum to maximum flow to the overall mean flow rate. We obtained a mean flow rate of 4.03 ± 0.17 mL/min (flow variation ~4.22%) in the above pressure range. As the inlet pressure was afterwards increased from 12 kPa to 17 kPa, the flow rate started to slowly increase, and the flow rate could not be maintained constant anymore in the test phase. According to the experimental results, although the valve only regulated the constant flow rate for the inlet pressures between 6 kPa to 12 kPa, it still showed a significant flow autoregulation capability when the pressure was higher than the minimum threshold pressure of 6 kPa. In order to validate the flow regulation capability of the valve, we fabricated and tested a straight through device (membraneless), and it outputted a continuously increasing flow rate in the whole test process. In comparison to the membrane valve, the flow rate of the device at the inlet pressure of 17 kPa increased 123.5% (Δ*Qs* in Figure 6a) against the flow rate at the pressure of 6 kPa, while the flow rate of the valve only increased by 23.7% (Δ*Qv* in Figure 6a) in the above pressure range. Thus, we could conclude that the valve was totally self-adaptive, and its flow resistance varied in correspondence with the inlet pressure to regulate the flow rate by the autonomous deflection of the membrane, thereby realizing a constant flow rate in a passive manner.

### 3.2. Flow Characterization Under Dynamic Pressure

It is necessary to investigate whether the valve is indeed capable of regulating flow rate through self-adaptive resistance variation when the inlet liquid pressure varies with the actuation time. To this objective, we examined the prototype valve under time-dependent varied pressures. The flow regulating performance of the prototype valve under different inlet pressures are shown in Figure 6b. The inlet pressures were varied in sinusoidal wave mode and square wave mode, respectively. Both the sinusoidal wave pressures and the square wave pressures fluctuated from 6 kPa to 12 kPa within a time period of 10 s, and the flow rates were recorded at every 0.01 s. The pressure range was determined for the constant flow phase under the static experiments. The mean flow rates under both pressure variations were 4 mL/min and 3.98 mL/min, respectively, which were quite close to those of the static pressure test. The standard deviation of the flow rate under sinusoidal wave pressures was 0.31 mL/min, which was equivalent to a flow variation of 7.75%. In the square wave pressure experiment, we found out that flow rate abruptly increased and decreased to give peak flow and valley points when the pressures were on high and low pulsations, respectively. We thought the behavior of the flow pulsation was directly influential to the response of the self-adaptive resistance variation of the valve. As described in the figure, the response time for the flow rate stabilization under the pulsating pressures was less than 0.3 s, and the standard deviation of the stable flow rate was 0.36 mL/min with the flow variation of 9.04%. Although the flow variations under the time-dependent pressures were much higher than in the static test, the valve still showed a great capability of regulating the flow. As the inlet pressure was increased by 100% (6 kPa to 12 kPa), the flow rates only increased by 16.8% under the sinusoidal condition and 19.9% under square wave. 

In comparison with the previously reported passive valves, the valve proposed in this work achieved a higher flow rate at a much lower threshold pressure, as shown in Table 1. This could be attributed to the special ellipsoid surface design incorporated in the control chamber. In most of the traditional membrane valves, the fluidic channel is designed with a rectangular cross-section which characteristically leaves a dead zone at the corners upon the deflection of the membrane. As the flow resistance of the valve should be increased to compensate the pressure increment, obtaining a required flow resistance quickly enough to compensate the pressure variations with such corners could be very difficult. To solve this problem, we designed an ellipsoid surface in the control chamber and two micro-sized holes in the membrane to allow liquid passage into the control chamber. The ellipsoidal surface design of the control chamber leaves quite an accommodating enclosure for the membrane to deflect into, thus facilitating a flow resistance development that could increase significantly, even under the low pressures. Furthermore, as the design in such of an inline configuration, liquid flows directly into the control chamber from the inlet, leaving no room for pressure loss in the valve, unlike in many traditional valves made up of exterior control channels. Therefore, the threshold pressure of our valve is less. In our study, we found the influence of the valve structure on the flow performance to be significant, precisely considering parameters such as diameter and depth of the ellipsoid surface of the control chamber, diameter of the liquid chamber, diameter and length of the outlet, thickness and Young’s modulus of the membrane, and diameter and location of the hole in the membrane. Further improvement on the flow performance of the valve could be achieved by optimizing the above parameters.

### 3.3. Low-Cost and Portable Gas-Driven Flow System

**Figure 7:**
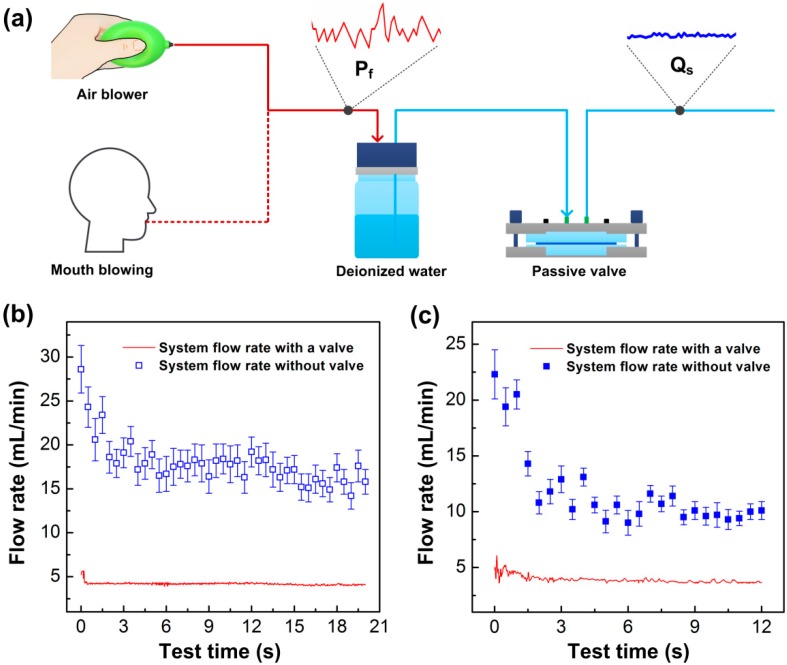
Application of the microfluidic passive valve for a stable flow in a low-cost portable gas-driven flow system. (**a**) Schematic diagram of the gas-driven flow system. Unstable pressure P_f_ was produced by an air blower and by human mouth to drive liquid in the system, and stable flow rate Q_s_ was achieved in the system. (**b**) Flow rate of the system operated by an air blower. (**c**) Flow rate of the system operated by mouth blown air.

For many small-scale biomedical applications, precise delivery of liquid at a low-cost manner is essential for the commercialization of these miniaturized devices and systems. As the commercial syringe pumps or peristaltic pumps are bulky in size while expensive in cost, it is necessary to develop a cost-effective small sized pump. Here, we came up with such a low-cost and portable gas-driven flow system which applied a microfluidic passive valve for accurate liquid control. The conceptualized system was composed of a pressure source, a sealed tank filled with deionized water and a microfluidic valve, shown in Figure 7a. The pressure source employed was that of a human mouth whose blown air pressure was considered enough to drive the liquid. To examine the flow stabilization capability of the system, a flow sensor and an electronic balance were used to measure the time-dependent flow rates as being regulated by the valve. Figure 7b shows the flow rates of the gas-driven flow system driven by the air blower. In the experiment, an operator held the air blower and slightly squeezed it to produce the pressurized air in the tank. The air pressure supplied by the air blower to the system was roughly in the range of 11.1 kPa to 14.2 kPa. The system was found to produce a stable flow rate after 0.5 s of actuation, and the mean flow rate of the system was 4.17 mL/min with a standard deviation of 0.23 mL/min, (or 5.52% of mean flow rate variation) across the time-varying pressures. For comparison, we also measured the flow rates of the system without the microfluidic valve, and the flow rate was 17.26 ± 3.1 mL/min with the flow variation of 17.96%. Figure 7c shows the flow performance of the gas-driven flow system driven by a human mouth. The air pressure produced by a strong mouth blowing was about 6.1 kPa to 8.2 kPa, which was a little higher than the minimum threshold pressure of the valve for flow stabilization. It was found that the flow rate of the system was regulated to be stable after about 2 s of actuation, and the mean flow rate of the stable liquid was 3.81 ± 0.24 mL/min (flow variation of 6.3%). The system without the valve driven by mouth blowing showed some dramatic flow fluctuations, with a mean flow rate of 10.44 ± 2.05 mL/min (19.64% variation). For both the air blower system and the mouth blowing system without the valve, the flow variations were far higher than in the valve incorporated systems, just as the figures clearly reveal. Therefore, we could conclude that the gas-driven flow system integrated with a passive valve was capable of achieving a stable flow rate. Hence, as the valve was able to maintain a constant flow under such a low threshold pressure, we can envision its usefulness in many microfluidic applications. For example, it can be applied to provide accurate sample fluids for high throughput cell sorting and concentration [40]. As the processing rate of cell separation and concentration can be several mL/min, a valve with the function of high throughput flow control will be very effective for the miniaturization of the microfluidic cell sorting system [36,37]. In addition, the valve structure can be further optimized to reduce the flow rate for more available microfluidic applications, such as sample mixing [41], droplet manipulation [42], drug delivery [43], etc.

## 4. Conclusions

In summary, we proposed a novel microfluidic passive valve for a stable flow control in microfluidic environment. The valve was made up of a control chamber of ellipsoid surface and an elastic membrane containing two micro-holes, which would deflect to change the flow resistance of the control chamber under the pressurized liquid flowing through the micro-holes, thereby maintaining a constant flow rate totally independent of the varied inlet pressures. To investigate the flow performances of the valve, we fabricated a prototype microvalve using 3D printing and UV laser cutting technologies, and the flow rates of the prototype were measured accordingly under static and dynamic inlet pressure conditions. The experimental results showed a high throughput and nearly constant flow rate achieved in the prototype under an ultra-low threshold pressure. To further examine the flow stability of the valve, we employed it in a portable gas-driven flow system which was operated by an air blower and human mouth blowing, respectively. The system was found to output a stable liquid under the low driving pressures, which validated the applicability of the valve in portable microfluidics.

## Figures and Tables

**Table 1:** Comparison of the different types of passive flow control valves.

Type	Constant Flow-Rate (mL/min)	Threshold Pressure (kPa)	Flow Variation (%)	Reference
Silicon membrane valve	0.022	20	5	[33]
Push-up valve	0.033	103	–	[34]
Planar check valve	1.2	100	3	[35]
Vertical parallel membrane valve	0.87	15	3.51	[32]
Horizontal parallel membrane valve	2.79	10	4.66	[36]
This work	4.03	6	4.22	–
